# Pharmacological thiamine levels as a therapeutic approach in Alzheimer's disease

**DOI:** 10.3389/fmed.2022.1033272

**Published:** 2022-10-04

**Authors:** Gary E. Gibson, Howard H. Feldman, Sheng Zhang, Sarah A. Flowers, José A. Luchsinger

**Affiliations:** ^1^Weill Cornell Medicine, Brain and Mind Research Institute, Burke Neurological Institute, White Plains, NY, United States; ^2^Alzheimer's Disease Cooperative Study and Department of Neurosciences, University of California, San Diego, San Diego, CA, United States; ^3^Proteomics and Metabolomics Facility, Institute of Biotechnology, Cornell University, Ithaca, NY, United States; ^4^Department of Neuroscience, Georgetown University, Washington, DC, United States; ^5^Departments of Medicine and Epidemiology, Columbia University Irving Medical Center, New York, NY, United States

**Keywords:** thiamine, Alzheimer's disease, benfotiamine, Advanced Glycation Endproducts (AGE), glucose metabolism, cognition, therapy

## Abstract

Summary of the study. 
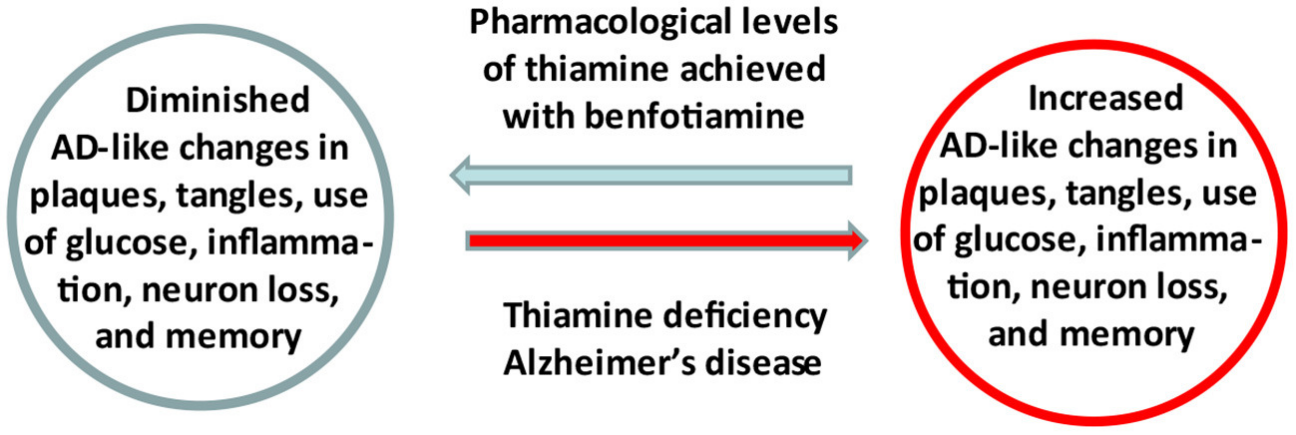

Evidence linking abnormalities in thiamine (vitamin B1) availability and metabolism to the pathophysiology of Alzheimer's Disease (AD) has focused attention on the regulation of thiamine as a therapeutic target. A recently completed pilot clinical trial in AD patients revealed that increasing blood thiamine to pharmacologically high levels using benfotiamine has potential efficacy in treating persons with early AD. These results support the underlying hypothesis that thiamine insufficiency promotes AD and is a druggable target. A mechanistic understanding of thiamine's cellular actions and improved methods to deliver thiamine to the brain are fundamental to optimize the use thiamine homeostasis as a target of engagement. Benfotiamine has a therapeutic product profile in AD that includes raising blood thiamine to pharmacologically high levels, with excellent safety and potential for clinical efficacy. It is a potentially widely available treatment.

The role of thiamine in memory/cognition and as the cause of the brain and memory disorder Wernicke-Korsakoff syndrome (WKS) has been well known since the 1930's (1), and data support a role in AD (2). Multiple batteries of memory tests demonstrate clinical similarities of AD and WKS (2). The activities of thiamine dependent enzymes are reduced in the brains of WKS patients (3) and in patients with AD (4, 5). Generally, the reductions in activities are not related to abundance in protein levels of relevant enzymes. This suggests that there is likely to be adequate enzyme, but inadequate thiamine availability (4, 6). We do not regard AD as WKS (e.g., the thiamine deficiency in WKS is caused by excessive alcohol intake). However, Wernicke syndrome with similar symptoms caused by thiamine deficiency is not necessarily associated with alcohol (7). Nevertheless, evidence suggests that there are mechanisms that may be involved in both diseases, and that thiamine deficiency is surprisingly common, particularly in the elderly, as reviewed below.

Experiments with thiamine deficient rodents support the hypothesis that thiamine is important in AD. Thiamine deficiency in normal and transgenic rodent models of AD leads to multiple AD like changes including: decreased brain glucose utilization (8), increased inflammation (9) and neuron loss (10), diminished cholinergic function (11) and exacerbated formation of plaques and tangles (12). Thiamine can prevent injury in Wernicke's syndrome, WKS and in animal models if thiamine is given before damage is irreversible (13). As discussed below, these observations stimulated studies to test whether pharmacologically high thiamine levels are beneficial in animal models of AD and/or in AD.

Brain thiamine deficiency can exist without nutritional thiamine deficits. Assessing thiamine status is difficult because individuals' requirements differ (14). The best method to assess thiamine status with blood measures is to determine the concentrations of thiamine, thiamine pyrophosphate (TPP) and thiamine monophosphate, as well as the TPP effect on transketolase activity (15–17). In tissues, activities transketolase and α-ketoglutarate dehydrogenase are good markers of thiamine status (13, 18). Multiple conditions can alter thiamine transport to the brain or its utilization or mobilization in or between cellular compartments without altering blood thiamine status. Conditions leading to tissue-level thiamine deficiency were the topics of a recent volume (19) and include: reduced thiamine intake (e.g., diet, gluten free diets, dialysis, celiac disease, bariatric surgery, excessive vomiting), increased thiamine excretion (e.g., diabetes), altered ability to use thiamine caused by at least 30 drugs, genetics (e.g., mutations in organic cation transporter 1 modulates multiple cardiometabolic traits through effects on thiamine content), and virus's (e.g., feline leukemia virus inhibits the thiamine transporter) (20) and liver cirrhosis (21). Indeed, the thiamine transporter declines with AD (22). High peripheral thiamine can overcome these abnormalities by increasing tissue thiamine availability.

Thiamine has many actions. TPP's critical role as a cofactor in key enzymes of the brain energy metabolism including transketolase (controls the pentose shunt), the pyruvate dehydrogenase complex [PDHC, links glycolysis to the tricarboxylic acid cycle (TCA)] and alpha ketoglutarate dehydrogenase complex (KGDHC, controls the TCA cycle) has been known for decades. Thiamine has many other actions including acting as an antioxidant, an anti-inflammatory, as a regulator of transcription etc. The non-coenzyme regulatory binding of thiamine and its esters has been demonstrated for the transcriptional regulator p53, poly(ADP-ribose) polymerase, prion protein PRNP, and a number of key metabolic enzymes that do not use TPP as a coenzyme (23, 24). The accumulated data indicate that the molecular mechanisms of the neurotropic action of thiamine are more complex than originally believed (24).

Optimal methods to increase brain thiamine. These observations stimulated trials of high-dose thiamine in AD in the 1990's (25, 26). While these trials were underpowered with a small number of patients they suggested some beneficial effects of high-dose thiamine (27). However, sustained higher thiamine levels cannot be achieved with thiamine in its usual preparations and the failure of thiamine treatment in previous studies can potentially be attributed to limitations of bioavailability. Pharmacokinetic and pharmacodynamic studies in humans and animals show that thiamine prodrugs such, as fursultiamine and benfotiamine, increase thiamine bioavailability much better than thiamine (28, 29). In an animal model of plaques, benfotiamine and fursultiamine increased blood thiamine about 150 and 50 times, respectively, and both elevate TMP and TDP about five-fold. While brain thiamine doubled, brain TMP and TPP were not altered. In the P301S mouse model of tangle formation, benfotiamine dramatically increased blood and liver thiamine with only modest effects in brain (about 20%) (30). In a Streptozotocin (STZ) model of AD, benfotiamine increased brain TPP about 50% (31).

Thiamine alters neurofilaments, a characteristic feature of AD. In non-transgenic mice, thiamine deficiency causes accumulation of neuritic clusters containing neurofilaments (10, 32, 33). In APP/PS1 transgenic mice, the thiamine deficiency induced abnormalities in neurites co-localized with APP-like protein and neurofilament (12). Thiamine deficiency induces a loss of axonal brain neurofilaments (34, 35). Benfotiamine/thiamine reverses the abnormal neurofilaments and neurites in tangles (30) or plaques (36).

Plaque formation is sensitive to thiamine levels in animal models. Thiamine deficiency exacerbates amyloid plaque pathology in Tg19959 transgenic mice, which over express a double mutant form of the amyloid precursor protein-APP. The area occupied by plaques in the cortex, hippocampus, and thalamus is enlarged by 50, 200, and 200%, respectively. Thiamine deficiency increases amyloid beta peptide1-42 levels by about three-fold and beta-secretase protein levels by 43% (12). Genetically reducing the thiamine dependent KGDHC by one half increases amyloid beta peptide and promotes plaque formation in mice (37). KGDHC dependent succinylation blocks alpha secretase and this block promotes Aβ42 plaque formation and amyloid beta aggregation (38). Free radical production due to reduced KGDHC activates beta secretase (37). The most compelling evidence of thiamine's role in plaque formation is that benfotiamine reduces plaque formation and improves memory in transgenic mice in a dose dependent manner (36).

Total tau and tau phosphorylation are sensitive to thiamine levels. Thiamine deficient humans with WKS syndrome have tangles (39, 40). In mice, thiamine deficiency increases phosphorylation of tau (12). Treatment with benfotiamine, diminishes phosphorylation of tau. This has been demonstrated in at least three different animal models of AD. Benfotiamine diminished the phosphorylation of tau in a mouse model bearing mutant human APP (33). In a mouse model of tangle formation (P301S), benfotiamine dose-dependently diminishes tangles (30), and improves behavioral outcomes (30, 36). Furthermore, a decline in transketolase activity in the P301S mice suggests that the brains of these transgenic mice are significantly thiamine deficient (30). This was rather surprising since tangles in these models are associated with this tau gene exon 10 mutation. Benfotiamine and diminishes phosphorylation of tau in the hippocampus in the streptozotocin (STZ) model of AD (31).

Compelling data links Advanced Glycation Endproducts (AGE) modifications to AD. AGE, also referred to as glycotoxins, are a diverse group of permanent carbohydrate modifications. They are created by the chemical addition of carbohydrates to proteins and lipids when glucose levels are not controlled in the cell (41, 42), a known risk factor for AD (43, 44). As AGE is persistent, AGE production is particularly problematic in the brain due to its slow protein turnover leading to accumulation of these toxins over the lifespan (45, 46). Further, there is a large group of crosslinking AGE which can form intra and inter-protein connections aggregating proteins (47), a hallmark of AD.

Elevated AGE and their receptor, RAGE, occur in the brain and periphery of AD patients and are found in both plaques and tangles (48–53). High concentrations of AGE are predictive of long-term decline in cognition-related daily living performance in patients with AD as measured by clinical dementia rating (CDR) or mini-mental status exam (MMSE) (40). Some AGE, such as pentosidine, a cross-linking AGE, have been shown to correlate with cognitive functioning in healthy individuals (53, 54). AGE have also been linked to *APOE* genotype, with *APOE4* carriers holding greater AGE compared to *APOE3* carriers, and the APOE4 molecule binding with greater affinity to AGE (55–59).

AGE are sensitive to thiamine deficiency and supplementation. Thiamine deficiency increases AGE, whereas elevating thiamine diminishes AGEs (60, 61). Even marginal thiamine deficiency increases AGE (61). Benfotiamine/thiamine diminishes AGE. The thiamine dependent enzyme transketolase can be activated by thiamine to reduce AGE (62). The activation of transketolase accelerates the precursors of AGEs toward the pentose phosphate pathway thereby reducing the production of AGE including carboxymethyllysine (CML) and pentosidine (63). In addition to activating transketolase, thiamine increases transcription of transketolase (64). A second well-established pathway for thiamine to diminish AGE is through the increased expression of the enzymes involved in the glyoxalase system, particularly glyoxalase 1 (GLO-1), which breaks down AGE precursors, primarily methylglyoxal (65). Since AGE including MG-H1 and carboxyethllysine (CEL) can be produced from methylglyoxal, the reduction in methylglyoxal by benfotiamine, reduces the AGE that are produced by this pathway. The most compelling evidence that interactions are potentially important to AD is that benfotiamine diminished AGE in parallel with encouraging clinical outcomes (66).

Thiamine also affects inflammation and microglial activity. Thiamine deficiency promotes inflammation whereas thiamine/benfotiamine diminish inflammation. Thiamine deficiency activates microglia (67, 68). Thiamine deficiency increases glial fibrillary acidic protein (GFAP) and inflammation in parallel with neuronal loss (33). Astrocytes as measured by GFAP are a major target of thiamine deficiency (9, 12). Thiamine deficiency in APP mutant mice increases GFAP expression and this parallels plaque formation (12) and dramatically increases brain p53 levels. Pro-inflammatory cytokines inhibit thiamine uptake (69). Thiamine/benfotiamine diminishes GFAP. In the STZ model of AD, benfotiamine reverses the inflammation, diminishes GFAP, and is protective (28). The action of thiamine as an anti-inflammatory factor has an important effect and has been shown to inhibit p53 intracellular activity, during re-replication and apoptosis (70). Thiamine's connection to inflammation is demonstrated by 12,500 references in google scholar.

These results stimulated a single site blinded Phase 2a randomized placebo-controlled pilot trial of benfotiamine to provide preliminary evidence of feasibility, safety, and efficacy. The trial tested whether a twelve-month treatment with benfotiamine would delay clinical decline in amyloid positron emission tomography (PET)- positive patients with amnestic mild cognitive impairment MCI (MMSE ≥ 26) or mild AD (26>MMSE>21) compared to placebo (52). The primary clinical outcome was Alzheimer's Disease Assessment Scale-Cognitive Subscale (ADAS-Cog-11) and secondary outcomes were the clinical dementia rating (CDR) score and brain glucose uptake measured by fluorodeoxyglucose (FDG)-PET. The trial showed that benfotiamine at a dose of 600 mg per day is safe and very well tolerated in patients with early AD. The treatment delivery achieved a 161-fold mean increase in blood thiamine. In the intent to treat population(ITT), the benfotiamine arm showed 43% reduction in the ADAS -Cog decline of the placebo group (*p* = 0.125), with a larger effect size in the CDR where the benfotiamine arm was 79.2% less than the decline in the placebo arm (*P* = 0.0129) (66).

Plasma measures from study participants revealed multiple metabolites/lipids as novel potential biomarkers that might be pharmacologically responsive to benfotiamine treatment. Two dozen biomarker candidates including thiamine, tyrosine, tryptophan, lysine, and 22 lipid species, mostly belonging to phosphatidylcholines reflected reversal of changes related to AD progression. The results suggest potential mechanistic pathways that underlie the benefit of benfotiamine in AD (71).

These encouraging results indicate the need for further research into the cellular mechanisms to optimize the treatment response as well as moving benfotiamine treatment into testing in larger, multicenter Proof of Concept clinical trials.

## Author contributions

All authors listed have made a substantial, direct, and intellectual contribution to the work and approved it for publication.

## Funding

The research was supported by NIH Grants 2P01AG014930 (GG and SZ), R01AG043679 (GG), K24AG045334 (JL), 1R01AG076634-01 (GG, JL, and HF), and R01AG072505 (SF). The Alzheimer Association (GG), Epstein Family Alzheimer's Disease Collaboration (HF).

## Conflict of interest

The authors declare that the research was conducted in the absence of any commercial or financial relationships that could be construed as a potential conflict of interest.

## Publisher's note

All claims expressed in this article are solely those of the authors and do not necessarily represent those of their affiliated organizations, or those of the publisher, the editors and the reviewers. Any product that may be evaluated in this article, or claim that may be made by its manufacturer, is not guaranteed or endorsed by the publisher.

## References

[1] Victor M Adams RD Collins GH. The Wernicke-Korsakoff syndrome. A clinical and pathological study of 245 patients, 82 with post-mortem examinations. Contemp Neurol Ser. (1971) 7:1–206. 5162155

[2] Kopelman MD. Frontal dysfunction and memory deficits in the alcoholic Korsakoff syndrome and Alzheimer-type dementia. Brain. (1991) 114A:117–37. 1998878

[3] Butterworth RF Kril JJ Harper CG. Thiamine-dependent enzyme changes in the brains of alcoholics: relationship to the Wernicke-Korsakoff syndrome. Alcohol Clin Exp Res. (1993) 17:1084–8. 10.1111/j.1530-0277.1993.tb05668.x 8279670

[4] Gibson GE Sheu K-FR Blass JP Baker A Carlson KC Harding B. Reduced activities of thiamine-dependent enzymes in the brains and peripheral tissues of patients with Alzheimer's disease. Arch Neurol. (1988) 45:836–40. 10.1001/archneur.1988.00520320022009 3395256

[5] Bubber P Haroutunian V Fisch G Blass JP Gibson GE. Mitochondrial abnormalities in Alzheimer brain: mechanistic implications. Ann Neurol. (2005) 57:695–703. 10.1002/ana.20474 15852400

[6] Mastrogiacomo F Lindsay JG Bettendorff L Rice J Kish SJ. Brain Protein and A-ketoglutarate dehydrogenase complex activity in Alzheimer-S disease. Ann Neurol. (1996) 39:592–8. 10.1002/ana.410390508 8619544

[7] Jolliffe N Wortis H Fein HD. The Wernicke syndrome. Arch Neurol Psychiatry. (1941) 46:569–97. 10.1001/archneurpsyc.1941.0228022000200125996397

[8] Hakim AM Carpenter S Pappius HM. Metabolic and histological reversibility of thiamine deficiency. J Cereb Blood Flow Metab. (1983) 3:468–77. 10.1038/jcbfm.1983.73 6630316

[9] Hazell AS Rao KVR Danbolt NC Pow DV Butterworth RF. Selective down-regulation of the astrocyte glutamate transporters Glt-1 and glast within the medial thalamus in experimental Wernicke's encephalopathy. J Neurochem. (2001) 78:560–8. 10.1046/j.1471-4159.2001.00436.x 11483659

[10] Ke Z-J Gibson GE. Selective response of various brain cell types during neurodegeneration induced by mild impairment of oxidative metabolism. Neurochem Int. (2004) 45:361–9. 10.1016/j.neuint.2003.09.008 15145550

[11] Barclay LL Gibson GE Blass JP. Impairment of behavior and acetylcholine metabolism in thiamine deficiency. J Pharmacol Exp Ther. (1981) 217:537. 7229989

[12] Karuppagounder SS Xu H Shi Q Chen LH Pedrini S Pechman D. Thiamine deficiency induces oxidative stress and exacerbates the plaque pathology in Alzheimer's mouse model. Neurobiol Aging. (2009) 30:1587–600. 10.1016/j.neurobiolaging.2007.12.013 18406011 PMC2782730

[13] Gibson GE Ksiezak-Reding H Sheu KFR Mykytyn V Blass JP. Correlation of enzymatic, metabolic, and behavioral deficits in thiamin deficiency and its reversal. Neurochem Res. (1984) 9:803–14. 10.1007/BF00965667 6149477

[14] Blass JP Gibson GE. Abnormality of a thiamine-requiring enzyme in patients with Wernicke-Korsakoff Syndrome. N Engl J Med. (1977) 297:1367–70. 10.1056/NEJM197712222972503 927453

[15] Whitfield KC Bourassa MW Adamolekun B Bergeron G Bettendorff L Brown KH. Thiamine deficiency disorders: diagnosis, prevalence, and a roadmap for global control programs. Ann N Y Acad Sci. (2018) 1430:3–43. 10.1111/nyas.13919 30151974 PMC6392124

[16] Jones KS Parkington DA Cox LJ Koulman A. Erythrocyte transketolase activity coefficient (Etkac) assay protocol for the assessment of thiamine status. Ann N Y Acad Sci. (2021) 1498:77–84. 10.1111/nyas.14547 33354793 PMC8451777

[17] Gallant J Chan K Green TJ Wieringa FT Leemaqz S Ngik R. Low-dose thiamin supplementation of lactating cambodian mothers improves human milk thiamine concentrations: a randomized controlled trial. Am J Clin Nutr. (2021) 114:90–100. 10.1093/ajcn/nqab052 33829271 PMC8246599

[18] Bunik VI Aleshin VA Zhou X Tabakov VY Karlsson A. Activation of mitochondrial 2-oxoglutarate dehydrogenase by cocarboxylase in human lung adenocarcinoma cells A549 Is P53/P21-dependent and impairs cellular redox state, mimicking the cisplatin action. Int J Mol Sci. (2020) 21:3759. 10.3390/ijms21113759 32466567 PMC7312097

[19] Gomes F Bergeron G Bourassa MW Fischer PR. Thiamine deficiency unrelated to alcohol consumption in high-income countries: a literature review. Ann N Y Acad Sci. (2021) 1498:46–56. 10.1111/nyas.14569 33576090 PMC8451800

[20] Mendoza R Miller AD Overbaugh J. Disruption of thiamine uptake and growth of cells by feline leukemia virus subgroup A. J Virol. (2013) 87:2412–9. 10.1128/JVI.03203-12 23269813 PMC3571393

[21] Lévy S Hervé C Delacoux E Erlinger S. Thiamine deficiency in hepatitis C virus and alcohol-related liver diseases. Dig Dis Sci. (2002) 47:543–8. 10.1023/A:1017907817423 11911339

[22] Ramamoorthy K Yoshimura R Al-Juburi S Anandam KY Kapadia R Alachkar A. Alzheimer's disease is associated with disruption in thiamin transport physiology: a potential role for neuroinflammation. Neurobiol Dis. (2022) 171:105799. 10.1016/j.nbd.2022.105799 35750148 PMC9744268

[23] Aleshin VA Mkrtchyan GV Bunik VI. Mechanisms of non-coenzyme action of thiamine: protein targets and medical significance. Biochemistry (Moscow). (2019) 84:829–50. 10.1134/S0006297919080017 31522667

[24] Mkrtchyan G Aleshin V Parkhomenko Y Kaehne T Luigi Di Salvo M Parroni A. Molecular mechanisms of the non-coenzyme action of thiamin in brain: biochemical, structural and pathway analysis. Sci Rep. (2015) 5:12583. 10.1038/srep12583 26212886 PMC4515825

[25] Blass JP Gleason P Brush D DiPonte P Thaler H. Thiamine and Alzheimer's disease. A pilot study. Arch Neurol. (1988) 45:833–5. 10.1001/archneur.1988.00520320019008 2969232

[26] Nolan KA Black RS Sheu KFR Langberg J Blass JP. A trial of thiamine in Alzheimer's disease. Arch Neurol. (1991) 48:81–3. 10.1001/archneur.1991.00530130093025 1986730

[27] Meador K Loring D Nichols M Zamrini E Rivner M Posas H. Preliminary findings of high-dose thiamine in dementia of Alzheimer's type. J Geriatr Psychiatry Neurol. (1993) 6:222–9. 10.1177/089198879300600408 8251051

[28] Sheng L Cao W Lin P Chen W Xu H Zhong C. Safety, tolerability and pharmacokinetics of single and multiple ascending doses of benfotiamine in healthy subjects. Drug Des Devel Ther. (2021) 15:1101–10. 10.2147/DDDT.S296197 33727798 PMC7955752

[29] Xie F Cheng Z Li S Liu X Guo X Yu P. Pharmacokinetic study of benfotiamine and the bioavailability assessment compared to thiamine hydrochloride. J Clin Pharmacol. (2014) 54:688–95. 10.1002/jcph.261 24399744

[30] Tapias V Jainuddin S Ahuja M Stack C Elipenahli C Vignisse J. Benfotiamine treatment activates the Nrf2/are pathway and is neuroprotective in a transgenic mouse model of tauopathy. Hum Mol Genet. (2018) 27:2874–92. 10.1093/hmg/ddy201 29860433 PMC6077804

[31] Moraes RCM Gonçalves ACd Portari GV Torrão AdS. Oral benfotiamine reverts cognitive deficit and increase thiamine diphosphate levels in the brain of a rat model of neurodegeneration. Exp Gerontol. (2020) 141:111097. 10.1016/j.exger.2020.111097 32987117

[32] Calingasan NY Gandy SE Baker H Sheu KFR Kim KS Wisniewski HM. Accumulation of amyloid precursor protein-like immunoreactivity in rat brain in response to thiamine deficiency. Brain Res. (1995) 677:50–60. 10.1016/0006-8993(95)00136-E 7606469

[33] Calingasan NY Gandy SE Baker H Sheu KF Smith JD Lamb BT. Novel neuritic clusters with accumulations of amyloid precursor protein and amyloid precursor-like protein 2 immunoreactivity in brain regions damaged by thiamine deficiency. Am J Pathol. (1996) 149:1063–71. 8780408 PMC1865137

[34] Peña CE Felter R. Ultrastructural changes of the lateral vestibular nucleus in acute experimental thiamine deficiency. Zeitschrift für Neurologie. (1973) 204:263–80. 10.1007/BF00316008 4804761

[35] Tellez I Terry RD. Fine structure of the early changes in the vestibular nuclei of the thiamine-deficient rat. Am J Pathol. (1968) 52:777–94. 5651225 PMC2013371

[36] Pan X Gong N Zhao J Yu Z Gu F Chen J. Powerful beneficial effects of benfotiamine on cognitive impairment and beta-amyloid deposition in amyloid precursor protein/presenilin-1 transgenic mice. Brain. (2010) 133(Pt 5):1342–51. 10.1093/brain/awq069 20385653

[37] Dumont M Ho DJ Calingasan NY Xu H Gibson G Beal MF. Mitochondrial dihydrolipoyl succinyltransferase deficiency accelerates amyloid pathology and memory deficit in a transgenic mouse model of amyloid deposition. Free Radic Biol Med. (2009) 47:1019–27. 10.1016/j.freeradbiomed.2009.07.008 19596066 PMC2761144

[38] Yang Y Tapias V Acosta D Xu H Chen H Bhawal R. Altered succinylation of mitochondrial proteins, app and tau in Alzheimer's disease. Nat Commun. (2022) 13:159. 10.1038/s41467-021-27572-2 35013160 PMC8748865

[39] Cullen KM Halliday GM. Neurof ibrillary tangles in chronic alcoholics. Neuropathol Appl Neurobiol. (1995) 21:312–8. 10.1111/j.1365-2990.1995.tb01065.x 7494599

[40] Cullen KM Halliday GM Caine D Kril JJ. The nucleus basalis (Ch4) in the alcoholic Wernicke-Korsakoff syndrome: reduced cell number in both amnesic and non-amnesic patients. J Neurol Neurosurg Psychiatry. (1997) 63:315. 10.1136/jnnp.63.3.315 9328247 PMC2169687

[41] Rabbani N Ashour A Thornalley PJ. Mass spectrometric determination of early and advanced glycation in biology. Glycoconj J. (2016) 33:553–68. 10.1007/s10719-016-9709-8 27438287 PMC4975772

[42] Soboleva A Vikhnina M Grishina T Frolov A. Probing protein glycation by chromatography and mass spectrometry: analysis of glycation adducts. Int J Mol Sci. (2017) 18:2557. 10.3390/ijms18122557 29182540 PMC5751160

[43] Luchsinger JA Reitz C Patel B Tang MX Manly JJ Mayeux R. Relation of diabetes to mild cognitive impairment. Arch Neurol. (2007) 64:570–5. 10.1001/archneur.64.4.570 17420320

[44] Biessels GJ Despa F. Cognitive decline and dementia in diabetes mellitus: mechanisms and clinical implications. Nat Rev Endocrinol. (2018) 14:591–604. 10.1038/s41574-018-0048-7 30022099 PMC6397437

[45] Kluever V Russo B Mandad S Kumar NH Alevra M Ori A. Protein lifetimes in aged brains reveal a proteostatic adaptation linking physiological aging to neurodegeneration. Sci Adv. (2022) 8:eabn4437. 10.1126/sciadv.abn4437 35594347 PMC9122331

[46] Dörrbaum AR Kochen L Langer JD Schuman EM. Local and global influences on protein turnover in neurons and glia. Elife. (2018) 7:e34202. 10.7554/eLife.34202 29914620 PMC6008053

[47] Chaudhuri J Bains Y Guha S Kahn A Hall D Bose N. The role of advanced glycation end products in aging and metabolic diseases: bridging association and causality. Cell Metab. (2018) 28:337–52. 10.1016/j.cmet.2018.08.014 30184484 PMC6355252

[48] Derk J MacLean M Juranek J Schmidt AM. The receptor for advanced glycation endproducts (Rage) and mediation of inflammatory neurodegeneration. J Alzheimers Dis Parkinsonism. (2018) 8:421. 10.4172/2161-0460.1000421 30560011 PMC6293973

[49] Chou PS Wu MN Yang CC Shen CT Yang YH. Effect of advanced glycation end products on the progression of Alzheimer's disease. J Alzheimers Dis. (2019) 72:191–7. 10.3233/JAD-190639 31561370

[50] Prasad K. Age–rage stress: a changing landscape in pathology and treatment of Alzheimer's disease. Mol Cell Biochem. (2019) 459:95–112. 10.1007/s11010-019-03553-4 31079281

[51] Haddad M Perrotte M Landri S Lepage A Fülöp T Ramassamy C. Circulating and extracellular vesicles levels of N-(1-Carboxymethyl)-L-Lysine (Cml) differentiate early to moderate Alzheimer's disease. J Alzheimers Dis. (2019) 69:751–62. 10.3233/JAD-181272 31127773

[52] Bär KJ Franke S Wenda B Müller S Kientsch-Engel R Stein G. Pentosidine and Nε-(Carboxymethyl)-Lysine in Alzheimer's disease and vascular dementia. Neurobiol Aging. (2003) 24:333–8. 10.1016/S0197-4580(02)00086-6 12498967

[53] Sharma A Weber D Raupbach J Dakal TC Fließbach K Ramirez A. Advanced glycation end products and protein carbonyl levels in plasma reveal sex-specific differences in Parkinson's and Alzheimer's disease. Redox Biol. (2020) 34:101546. 10.1016/j.redox.2020.101546 32460130 PMC7251371

[54] Spauwen PJ van Eupen MG Köhler S Stehouwer CD Verhey FR van der Kallen CJ. Associations of advanced glycation end-products with cognitive functions in individuals with and without type 2 diabetes: the Maastricht Study. J Clin Endocrinol Metab. (2015) 100:951–60. 10.1210/jc.2014-2754 25459912

[55] Li YM Dickson DW. Enhanced binding of advanced glycation endproducts (Age) by the Apoe4 isoform links the mechanism of plaque deposition in Alzheimer's disease. Neurosci Lett. (1997) 226:155–8. 10.1016/S0304-3940(97)00266-8 9175590

[56] Chen J Mooldijk SS Licher S Waqas K Ikram MK Uitterlinden AG. Assessment of advanced glycation end products and receptors and the risk of dementia. JAMA Netw Open. (2021) 4:e2033012. 10.1001/jamanetworkopen.2020.33012 33416887 PMC7794665

[57] Deo P Dhillon VS Chua A Thomas P Fenech M. Apoe E4 carriers have a greater propensity to glycation and srage which is further influenced by rage G82s polymorphism. J Gerontol A Biol Sci Med Sci. (2020) 75:1899–905. 10.1093/gerona/glz259 31677348

[58] Lambert JC Ibrahim-Verbaas CA Harold D Naj AC Sims R Bellenguez C. Meta-analysis of 74,046 individuals identifies 11 new susceptibility loci for Alzheimer's disease. Nat Genet. (2013) 45:1452–8. 10.1038/ng.2802 24162737 PMC3896259

[59] Mooldijk SS Chen J Ikram MA Zillikens MC. Letter to the Editor, Reacting To: “Apoe E4 carriers have a greater propensity to glycation and srage which is further influenced by rage G82s polymorphism”. J Gerontol A Biol Sci Med Sci. (2020) 75:1906–7. 10.1093/gerona/glaa037 32009162 PMC7518568

[60] Alkhalaf A Kleefstra N Groenier KH Bilo HJG Gans ROB Heeringa P. Effect of benfotiamine on advanced glycation endproducts and markers of endothelial dysfunction and inflammation in diabetic nephropathy. PLoS ONE. (2012) 7:e40427. 10.1371/journal.pone.0040427 22792314 PMC3391239

[61] Depeint F Bruce WR Shangari N Mehta R O'Brien PJ. Mitochondrial function and toxicity: role of the B vitamin family on mitochondrial energy metabolism. Chem Biol Interact. (2006) 163:94–112. 10.1016/j.cbi.2006.04.014 16765926

[62] Hammes HP Du X Edelstein D Taguchi T Matsumura T Ju Q. Benfotiamine blocks three major pathways of hyperglycemic damage and prevents experimental diabetic retinopathy. Nat Med. (2003) 9:294–9. 10.1038/nm834 12592403

[63] Stracke H Gaus W Achenbach U Federlin K Bretzel RG. Benfotiamine in diabetic polyneuropathy (Bendip): results of a randomised, double blind, placebo-controlled clinical study. Exp Clin Endocrinol Diabetes. (2008) 116:600–5. 10.1055/s-2008-1065351 18473286

[64] Pekovich SR Martin PR Singleton CK. Thiamine deficiency decreases steady-state transketolase and pyruvate dehydrogenase but Not A-ketoglutarate dehydrogenase Mrna levels in three human cell types. J Nutr. (1998) 128:683–7. 10.1093/jn/128.4.683 9521628

[65] He Y Zhou C Huang M Tang C Liu X Yue Y. Glyoxalase system: a systematic review of its biological activity, related-diseases, screening methods and small molecule regulators. Biomed Pharmacother. (2020) 131:110663. 10.1016/j.biopha.2020.110663 32858501

[66] Gibson GE Luchsinger JA Cirio R Chen H Franchino-Elder J Hirsch JA. Benfotiamine and cognitive decline in Alzheimer's disease: results of a randomized placebo-controlled phase iia clinical trial. J Alzheimers Dis. (2020) 78:1−22. 10.3233/JAD-200896 33074237 PMC7880246

[67] Ke Z-J Degiorgio LA Volpe BT Gibson GE. Reversal of thiamine deficiency-induced neurodegeneration. J Neuropathol Exp Neurol. (2003) 62:195–207. 10.1093/jnen/62.2.195 12578229

[68] Calingasan NY Chun WJ Park LCH Uchida K Gibson GE. Oxidative stress is associated with region-specific neuronal death during thiamine deficiency. J Neuropathol Exp Neurol. (1999) 58:946–58. 10.1097/00005072-199909000-00005 10499437

[69] Anandam KY Srinivasan P Yasujima T Al-Juburi S Said HM. Proinflammatory cytokines inhibit thiamin uptake by human and mouse pancreatic acinar cells: involvement of transcriptional mechanism (S). Am J Physiol Gastrointest Liver Physiol. (2021) 320:G108–G16. 10.1152/ajpgi.00361.2020 33146542 PMC8112188

[70] Manzetti S Zhang J van der Spoel D. Thiamin function, metabolism, uptake, and transport. Biochemistry. (2014) 53:821–35. 10.1021/bi401618y 24460461

[71] Bhawal R Fu Q Anderson ET Gibson GE Zhang S. Serum metabolomic and lipidomic profiling reveals novel biomarkers of efficacy for benfotiamine in Alzheimer's disease. Int J Mol Sci. (2021) 22:13188. 10.3390/ijms222413188 34947984 PMC8709126

